# Polygenic risk for type 2 diabetes, lifestyle, metabolic health, and cardiovascular disease: a prospective UK Biobank study

**DOI:** 10.1186/s12933-022-01560-2

**Published:** 2022-07-14

**Authors:** Jae-Seung Yun, Sang-Hyuk Jung, Manu Shivakumar, Brenda Xiao, Amit V. Khera, Hong-Hee Won, Dokyoon Kim

**Affiliations:** 1 Department of Biostatistics, Epidemiology and Informatics, Perelman School of Medicine, University of Pennsylvania, B304 Richards Building, 3700 Hamilton Walk, Philadelphia, PA 19104-6021 USA; 2 Division of Endocrinology and Metabolism, Department of Internal Medicine, College of Medicine, St. Vincent’s Hospital, The Catholic University of Korea, Seoul, Republic of Korea; 3 Department of Digital Health, SAIHST, Sungkyunkwan University, Samsung Medical Center, Seoul, Republic of Korea; 4 Institute for Biomedical Informatics, University of Pennsylvania, Philadelphia, PA USA; 5 Genomics and Computational Biology Graduate Group, University of Pennsylvania, Philadelphia, PA USA; 6 Center for Genomic Medicine, Massachusetts General Hospital, Boston, MA USA; 7 Samsung Advanced Institute for Health Sciences and Technology (SAIHST), Samsung Medical Center, Sungkyunkwan University, 81 Irwon-ro, Gangnam-gu, Seoul, 06351 Republic of Korea; 8 Samsung Genome Institute, Samsung Medical Center, Sungkyunkwan University School of Medicine, Seoul, Republic of Korea

**Keywords:** Polygenic risk score, Lifestyle, Metabolic health, Cardiovascular disease, Type 2 diabetes

## Abstract

**Background:**

Few studies have examined associations between genetic risk for type 2 diabetes (T2D), lifestyle, clinical risk factors, and cardiovascular disease (CVD). We aimed to investigate the association of and potential interactions among genetic risk for T2D, lifestyle behavior, and metabolic risk factors with CVD.

**Methods:**

A total of 345,217 unrelated participants of white British descent were included in analyses. Genetic risk for T2D was estimated as a genome-wide polygenic risk score constructed from > 6 million genetic variants. A favorable lifestyle was defined in terms of four modifiable lifestyle components, and metabolic health status was determined according to the presence of metabolic syndrome components.

**Results:**

During a median follow-up of 8.9 years, 21,865 CVD cases (6.3%) were identified. Compared with the low genetic risk group, participants at high genetic risk for T2D had higher rates of overall CVD events, CVD subtypes (coronary artery disease, peripheral artery disease, heart failure, and atrial fibrillation/flutter), and CVD mortality. Individuals at very high genetic risk for T2D had a 35% higher risk of CVD than those with low genetic risk (HR 1.35 [95% CI 1.19 to 1.53]). A significant gradient of increased CVD risk was observed across genetic risk, lifestyle, and metabolic health status (*P* for trend > 0.001). Those with favorable lifestyle and metabolically healthy status had significantly reduced risk of CVD events regardless of T2D genetic risk. This risk reduction was more apparent in young participants (≤ 50 years).

**Conclusions:**

Genetic risk for T2D was associated with increased risks of overall CVD, various CVD subtypes, and fatal CVD. Engaging in a healthy lifestyle and maintaining metabolic health may reduce subsequent risk of CVD regardless of genetic risk for T2D.

**Supplementary Information:**

The online version contains supplementary material available at 10.1186/s12933-022-01560-2.

## Background

Cardiovascular disease (CVD) is one of the leading causes of mortality, and has become a global public health concern [1]. As such, it is of particular importance to document cardiovascular (CV) risk factors and to establish effective strategies for prevention of CV complications. Both lifestyle and clinical metabolic risk factors play important roles in the complex mechanism of CVD. Studies have consistently reported CVD risk to be associated with lifestyle factors including obesity, smoking, insufficient physical activity, and unhealthy eating habits [2]. Metabolic syndrome (MetS) is a cluster of clinical factors that increase the risk of chronic metabolic diseases such as type 2 diabetes (T2D) and CVD [3]. Since MetS components can manifest during the asymptomatic preclinical period, their management can be an effective preventive strategy for CVD, as can lifestyle modification [4].

Despite recent advances in management of CV risk factors, CVD remains a serious complication of T2D [4]. Population-based epidemiologic studies have confirmed T2D and CVD to share many clinical risk factors, and hyperglycemia is itself significantly related to increased risk of CVD and CV mortality [5]. Moreover, individuals with T2D have two- to threefold increased risk of CV mortality over healthy individuals [6]. Recent advances in genome-wide association studies (GWAS) have provided more evidence for the relationship between diabetes and CVD from a genomic point of view, with several studies suggesting the diseases to share genetic components and genetic correlation [7, 8]. In addition, Mendelian randomization (MR) studies have demonstrated that genetic variants associated with fasting glucose, insulin resistance, and T2D have causal relationships with coronary heart disease and ischemic stroke [9, 10].

Over the last decade, the growth of large consortia and biobanks has enabled identification of numerous genetic variants associated with complex diseases. In addition, recent advances in statistical analytical approaches have facilitated the quantification of genetic risk for a specific disease. One such method is the polygenic risk score (PRS), which aggregates millions of common variants (predominantly single-nucleotide polymorphisms, SNPs), weighted by the impact of each allele on disease risk, into a simplified score; this method has emerged as a useful genetic marker for common diseases such as coronary artery disease (CAD) or T2D [11]. However, no studies to date have evaluated the association of genetic risk for T2D with risk of developing CVD. Furthermore, the development and progression of T2D and CVD alike are known to be driven by complex interactions between genetic predisposition and lifestyle acting on metabolic health status [12]. A previous study has shown that T2D PRS and lifestyle habits together have additive deleterious effects on T2D risk [13]. However, little is known about the interactions between genetic predisposition to T2D, lifestyle, and metabolic health in the context of CVD risk.

In this study, we aimed to investigate the association of genetic risk for T2D with subsequent risk of CVD using a T2D PRS constructed from more than six million SNPs genotyped in individuals participating in the UK Biobank study. We further examined the beneficial reduction in CVD risk provided by two modifiable factors, lifestyle habits and metabolic health profile, across groups stratified by genetic risk for T2D. We also examined whether the magnitude of the association between T2D PRS and CVD varies by age group.

## Materials and methods

### Study population

The UK Biobank is a large prospective observational cohort study that has recruited > 500,000 adults across 22 centers located throughout the United Kingdom. The full protocol of the UK Biobank study is publicly available, and the study design and measurement methods have been described elsewhere [14]. Participants aged 40–69 years were enrolled between 2006 and 2010 and were followed up with for subsequent health events. We excluded participants who had a prior history of CAD, ischemic stroke, hemorrhagic stroke, peripheral artery disease (PAD), atrial fibrillation/flutter, or heart failure (HF) at baseline (n = 42,633).

The UK Biobank was approved by the National Research Ethics Committee (June 17, 2011, extended on May 10, 2016 [RES reference 16/NW/0274]). Participants provided written informed consent allowing use of their samples and data for medical research purposes. The present research using the UK Biobank Resource was approved under Application Number 67855.

### Genotyping and quality control

UK Biobank samples (version 3; March 2018) were genotyped for > 800,000 SNPs using either the Affymetrix UK BiLEVE Axiom array or the Affymetrix UK Biobank Axiom array. Imputation via IMPUTE2 was carried out centrally by UK Biobank researchers using the merged 1000 Genomes Project panel and UK 10K panel [15]. After imputation, variant-level quality control (QC) was performed by filtering SNPs on two criteria: (1) minor allele frequency < 0.01 and (2) imputation quality score < 0.3. A total of 9,505,768 imputed autosomal SNPs passed the QC criteria. Sample-level QC was performed by excluding samples on the basis of (1) participants identified as not of ‘White-British’ ancestry according to either self-report or principal component (PC) analysis of genetic ancestry, (2) mismatched sex, or (3) having second-degree or closer relatives also in the Biobank. After exclusion, 345,217 White-British participants were determined eligible for the genetic analyses.

### Polygenic risk scores

The T2D and CAD PRSs utilized in this study were respectively derived from the DIAGRAM (DIAbetes Genetics Replication And Meta-analysis) [16] and the CARDIOGRAMplusC4D (Coronary Artery Disease Genome wide Replication and Meta-analysis plus the Coronary Artery Disease Genetics) consortium [17], and used pre-calculated weights previously derived by Khera et al. [11] with LDpred [18]. To determine the PRSs for each individual, we calculated the aggregated risk score as the weighted sum of risk alleles using PLINK 1.90 [19] and the beta coefficients.

### Ascertainment of CVD outcomes

The primary outcome for our study was incident CVD, defined as the time to the first occurrence of a composite event inclusive of CAD, ischemic stroke, hemorrhagic stroke, PAD, HF, and atrial fibrillation/flutter. Cases of incident CVD were ascertained based on the recorded first occurrence of disease and on hospitalization records. Detailed information regarding CVD outcome is provided in Additional file 1: Table S1.

### Ascertainment of variables

At enrollment, information on participants’ sociodemographic characteristics, health and medical history, and lifestyle factors was collected using a self-administered touchscreen questionnaire and in-person baseline interviews. During the interviews, height, body weight, waist, and hip circumference were measured by trained staff using standardized procedures.

According to the guidelines of the American Heart Association (AHA), four factors are considered to primarily define lifestyle behaviors: current smoking, obesity, physical activity, and eating habits [20, 21]. Smoking status was classified as current smoker or non-smoker. Obesity was defined as a body-mass index (BMI) ≥ 30 kg/m^2^ according to the World Health Organization international classification. With respect to physical activity, participants were classified as having a healthy lifestyle if they reported more than 5 days per week of moderate activity or vigorous activity. Eating habits were defined following recommendations on dietary priorities for CV health, which categorize common diet components as fruits, vegetables, whole grains, fish, dairy, refined grains, processed meats, unprocessed meats, and sugar-sweetened beverages. Eating habits were considered healthy if participants adhered to at least half of the dietary recommendations for CV health, as assessed by a food frequency questionnaire [22]. Collectively, lifestyle behaviors were categorized into three groups: unfavorable (0-1 healthy lifestyle factor) [1], intermediate (2 healthy lifestyle factors), and favorable (≥ 3 healthy lifestyle factors). Metabolic health status was identified according to the presence of the five components of MetS, based on criteria from the IDF consensus report [23]. Detailed definitions of lifestyle habits and metabolic health status are described in Additional file 1: Table S2.

During the baseline assessment visit, blood samples were obtained and processed according to standardized protocols [24]. The procedures for sampling and processing blood and urine samples have been described previously [25]. HbA1c was determined by high performance liquid chromatography using the Bio-Rad Variant II Turbo Analyzer. Glucose, lipid profiles, and inflammatory markers were determined using the Beckman Coulter AU5800 with the following assays: hexokinase analysis for HbA1c, CHO-POD analysis for total cholesterol, GPO-POD analysis for triglycerides, enzyme immunoinhibition analysis for high-density lipoprotein (HDL) cholesterol, enzymatic selective protection analysis for low-density lipoprotein (LDL) cholesterol, and immunoturbidimetric assays for lipoprotein (a) and high sensitivity C-reactive protein. More details regarding serum biomarker data are available on the UK Biobank website at https://www.ukbiobank.ac.uk.

Information regarding major comorbidities was obtained from (1) the self-report collected via in-person interview or touchscreen questionnaire at enrollment, (2) diagnostic or procedure codes in the electronic health records database linked to hospital admission records, and (3) the first occurrence of the comorbidity in the health outcomes database, which is linked with hospital in-patient records, death records, cancer registry, and primary care records (Additional file 1: Table S3). The Charlson Comorbidity Index (CCI) was calculated based on diagnostic codes [26]. Date and cause of death were extracted from death certificates held by the NHS Information Centre (England and Wales) and the NHS Central Register (Scotland).

### Statistical analysis

The demographic characteristics of cases and non-cases were evaluated for difference using chi-square tests for categorical variables or independent *t*-tests for continuous variables. The incidence rate of CVD events is presented along with the rate of events per 1,000 person years. The associations of genetic risk, lifestyle, and metabolic health with CVD were evaluated using Cox proportional hazard regression models. Hazard ratios (HRs) of PRSs for CVD were used both as quantitative variables reported per one standard deviation (SD) and categorical variables defined as follows: low (0–19th percentile), intermediate (20–79th percentile), high (80–98th percentile), and very high (99th percentile). We considered the top 1% of the PRS distribution as a very high-risk group in light of the curve of cumulative incidence of prevalent disease over the PRS distribution (Additional file 1: Fig. S1). Multivariable Cox regression analyses were used to assess the main association with adjustment for age, sex, genotyping array, and the first ten PCs of genetic ancestry as confounding factors. The main association was also adjusted for Townsend deprivation index, income level, baseline blood pressure, lifestyle behavior, laboratory findings, CCI, and major comorbidities including hypertension, dyslipidemia, cancer, chronic liver disease, chronic lung disease, and chronic kidney disease.

First, we analyzed the association between T2D PRS risk group and subsequent risk for overall CVD and for each CVD subtype, taking the T2D PRS low-risk group as the reference group. Second, we assessed the interaction of genetic risk with lifestyle habits, genetic risk with metabolic health, and age with subsequent risk of overall CVD. To test for multiplicative interactions, we included two-factor interaction terms in the Cox regression models and tested their significance using likelihood ratios. Considering the sample size and associated statistical power, before proceeding with the interaction analysis including all factors of genetic risk, lifestyle, and metabolic profile, we first recategorized participants into two or three groups for each of the three aspects being considered: low and high genetic risk (bottom 80 percentile vs. top 20 percentile for T2D PRS), favorable and non-favorable lifestyle (favorable vs. intermediate or unfavorable), and metabolically healthy and unhealthy status (1 or less vs. 2 vs. 3 or more MetS components). We also conducted sensitivity analyses using different genetic risk categories to avoid an ad-hoc categorization. All analyses for the association between genetic risk, lifestyle, and metabolic health were adjusted for age, sex, genotyping array, and the first ten PCs of genetic ancestry.

Individuals were censored at the date of follow-up loss, the date of follow-up end (January 31, 2018 for England and Wales; November 30, 2016 for Scotland), or the date of death, whichever came first. Individuals with missing data were excluded from each model (Additional file 1: Table S3). Schoenfeld residuals and log minus log plots were used to assess the proportional hazard assumption. All statistical tests were two-sided, and *P* < 0.05 was defined as statistically significant. All statistical analyses were performed using PLINK 1.9 [19] and R (version 3.9.0).

## Results

### Population characteristics

In total, 345,217 participants who did not have prior CVD history were included in this study. The mean age of participants was 56.1 years, and 44.6% were men. Participants who had higher genetic risk for T2D exhibited higher prevalence of T2D and major chronic comorbidities except for cancer. Participants who had higher genetic risk for T2D were younger, had poor metabolic health profiles, and adhered to unfavorable lifestyles. Baseline demographics and clinical characteristics are shown in Table 1.

**Table 1:** Baseline characteristics of the study population

	Total	T2D PRS				
		Low	Intermediate	High	Very high	*P* value
		0–19th percentile	20–79th percentile	80–98th percentile	99th percentile	
	(n = 345,217)	(n = 69,070)	(n = 207,192)	(n = 65,505)	(n = 3450)	
*Demographics & physical measurement*						
Age (years)	56.1 ± 8.0	56.2 ± 7.9	56.0 ± 8.0	56.0 ± 8.0	55.8 ± 7.9	< 0.001
Male	153,959 (44.6)	30,870 (44.7)	92,409 (44.6)	29,127 (44.5)	1553 (45.0)	0.809
Systolic blood pressure (mmHg)	140.2 ± 19.6	139.4 ± 19.7	140.2 ± 19.6	141.0 ± 19.6	142.1 ± 19.3	< 0.001
Diastolic blood pressure (mmHg)	82.5 ± 10.6	82.0 ± 10.6	82.5 ± 10.6	83.0 ± 10.6	83.7 ± 10.5	< 0.001
BMI (kg/m^2^)	27.3 ± 4.7	26.9 ± 4.5	27.3 ± 4.7	27.7 ± 4.8	28.3 ± 5.1	< 0.001
Waist circumference (cm)	89.8 ± 13.3	88.7 ± 13.1	89.8 ± 13.3	90.8 ± 13.4	92.5 ± 13.6	< 0.001
Townsend deprivation index	− 1.62 ± 2.90	− 1.65 ± 2.89	− 1.62 ± 2.89	− 1.61 ± 2.91	− 1.53 ± 2.87	0.003
*Income level*						< 0.001
Less than 18,000£	61,532 (20.6)	11,952 (20.0)	37,084 (20.7)	11,858 (21.0)	638 (21.6)	
18,000 to 30,999£	76,307 (25.6)	15,166 (25.4)	45,831 (25.6)	14,556 (25.8)	754 (25.5)	
31,000 to 51,999£	80,527 (27.0)	16,135 (27.0)	48,344 (27.0)	15,237 (27.0)	811 (27.4)	
52,000 to 100,000£	63,331 (21.2)	13,046 (21.9)	37,916 (21.2)	11,740 (20.8)	629 (21.2)	
Greater than 100,000£	16,347 (5.5)	3404 (5.7)	9748 (5.4)	3067 (5.4)	128 (4.3)	
*Lifestyle and metabolic health*						
Lifestyle habits						< 0.001
Favorable	182,991 (55.0)	38,009 (57.1)	109,760 (55.0)	33,562 (53.2)	1660 (50.2)	
Intermediate	111,276 (33.4)	21,769 (32.7)	66,812 (33.5)	21,512 (34.1)	1183 (35.8)	
Unfavorable	38,450 (11.6)	6805 (10.2)	23,110 (11.6)	8069 (12.8)	466 (14.1)	
Number of metabolic syndrome components						< 0.001
0 component	42,022 (14.0)	9652 (16.0)	25,187 (14.0)	6889 (12.1)	294 (9.8)	
1 component	93,323 (31.1)	20,157 (33.5)	56,053 (31.1)	16,326 (28.7)	787 (26.3)	
2 components	81,314 (27.1)	15,980 (26.6)	48,955 (27.2)	15,619 (27.4)	760 (25.4)	
3 or more components	83,448 (27.8)	14,390 (23.9)	49,761 (27.7)	18,142 (31.8)	1155 (38.6)	
*Laboratory findings*						
HbA1c (%)	5.4 ± 0.6	5.4 ± 0.5	5.4 ± 0.6	5.5 ± 0.6	5.6 ± 0.8	< 0.001
Fasting plasma glucose (mg/dL)	91.7 ± 20.6	90.4 ± 17.1	91.6 ± 20.3	93.3 ± 23.9	96.0 ± 30.3	
estimated GFR (ml/min/1.73m^2^)	79.0 ± 14.0	78.9 ± 13.8	79.0 ± 14.0	79.2 ± 14.2	79.4 ± 14.5	< 0.001
Total cholesterol (mg/dL)	223.7 ± 42.9	223.9 ± 42.0	223.7 ± 42.9	223.5 ± 43.8	223.3 ± 45.6	0.168
Triglyceride (mg/dL)	154.5 ± 90.0	147.4 ± 85.4	154.3 ± 89.6	161.8 ± 94.4	171.0 ± 100.4	< 0.001
HDL-cholesterol (mg/dL)	56.6 ± 14.7	57.6 ± 14.8	56.7 ± 14.7	55.6 ± 14.5	54.3 ± 14.6	< 0.001
LDL-cholesterol (mg/dL)	140.1 ± 32.8	139.9 ± 32.2	140.1 ± 32.8	140.3 ± 33.4	140.5 ± 34.3	0.014
*Baseline major comorbidity*						
Type 2 diabetes	11,372 (3.5)	1274 (1.9)	6646 (3.4)	3210 (5.2)	242 (7.5)	< 0.001
Dyslipidemia	48,196 (14.0)	8461 (12.2)	28,774 (13.9)	10,343 (15.8)	618 (17.9)	< 0.001
Hypertension	90,707 (26.3)	16,180 (23.4)	54,454 (26.3)	18,926 (28.9)	1147 (33.2)	< 0.001
Chronic lung disease	49,037 (14.2)	9266 (13.4)	29,629 (14.3)	9618 (14.7)	524 (15.2)	< 0.001
Chronic kidney disease	4114 (1.2)	732 (1.1)	2484 (1.2)	834 (1.3)	64 (1.9)	< 0.001
Chronic liver disease	3446 (1.0)	635 (0.9)	2032 (1.0)	730 (1.1)	49 (1.4)	< 0.001
Cancer	40,497 (11.7)	8171 (11.8)	24,219 (11.7)	7711 (11.8)	396 (11.5)	0.877
Charlson comorbidity index	0.48 ± 0.86	0.46 ± 0.85	0.48 ± 0.86	0.51 ± 0.88	0.54 ± 0.90	< 0.001

Data are n (%) or mean (SD)

BMI: body-mass index; HDL: high-density lipoprotein; LDL: low-density lipoprotein; T2D: type 2 diabetes; PRS: polygenic risk score

### Association of T2D PRS with CVD outcome

**Fig. 1:**
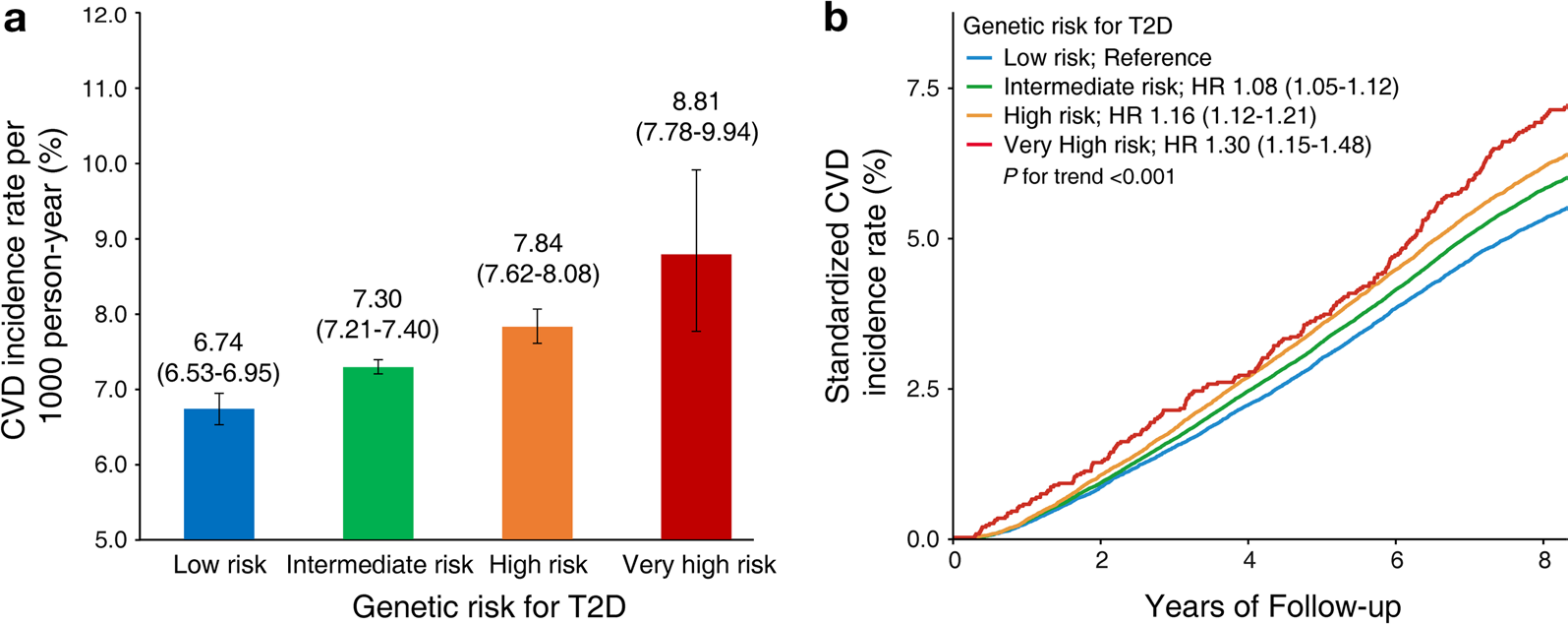
**A** CVD incidence rates according to the risk groups of T2D PRS. **B** Hazard plot for CVD risk according to the risk group of T2D PRS. T2D PRS risk groups: low (0–19th percentile), intermediate (20–79th percentile), high (80–98th percentile), and very high (99th percentile). Error bars represent 95% CI of estimated cumulative incidence. CVD: cardiovascular disease; T2D: type 2 diabetes; PRS: polygenic risk score; HR: hazard ratio; CI: confidence interval

**Fig. 2:**
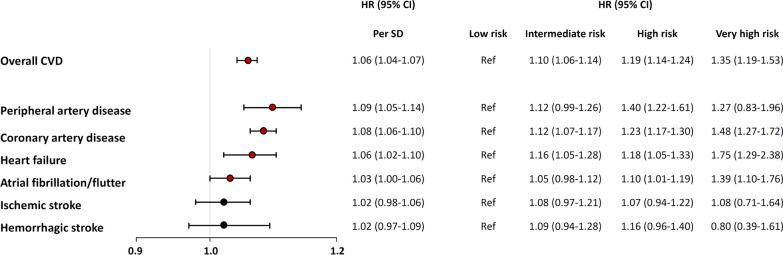
Hazard ratio for overall and subtypes of cardiovascular outcome according to T2D PRS risk group. CVD: cardiovascular disease; T2D: type 2 diabetes; PRS: polygenic risk score; HR: hazard ratio; CI: confidence interval; SD: standard deviation

During the median 8.9-year (Interquartile range 8.3 to 9.5) follow-up period, we documented 21,865 (6.3%) instances of incident CVD, giving an overall incidence rate of 7.30 events per 1000 person-years (95% confidence interval (CI) 7.21–7.40). Compared to the group with lower T2D PRS, those with higher genetic risk exhibited a higher absolute incidence rate of CVD (Fig. 1, Additional file 1: Fig. S2). In the Cox regression analysis, T2D PRS was significantly associated with subsequent risk of CVD (HR 1.06 [95% CI 1.04–1.07] per one SD, *P* < 0.001). Of CVD outcomes, the strongest risk was observed for PAD; CAD, atrial fibrillation, and HF also showed significantly increased risk as PRS for T2D increased. On the other hand, there was no significant relationship of T2D PRS with ischemic stroke or hemorrhagic stroke (Fig. 2). Notably, the significant association of higher genetic risk of T2D with risk of CVD persisted even after adjustment for socioeconomic factors, baseline blood pressure, BMI, lifestyle behaviors, T2D, and other major comorbidities (Additional file 1: Tables S4–5). However, this association was attenuated after further adjustment for HbA1c, lipid profiles, renal function, and C-reactive protein. We further related T2D PRS with the PRS for CAD, which constituted the largest portion of CVD events in this study. The two scores exhibited a Pearson correlation coefficient of 0.062, and the concordance among risk groups was 44.5% (Additional file 1: Fig. S3, Additional file 1: Table S6). Notably, after adjustment for CAD PRS, the risk of CVD was still significantly increased in those participants having high genetic risk for T2D (Additional file 1: Table S5, model 3).

In total, 1963 (0.6%) participants died due to CVD during the follow-up period. Compared to those at low genetic risk, participants with higher genetic risk for T2D had a higher rate of CV mortality (Additional file 1: Table S7), and those having very high genetic risk had 1.6-fold increased risk of CV mortality (Additional file 1: Table S8).

### Influence of T2D PRS, lifestyle behaviors, and metabolic health on CVD risk

Compared with participants who had low genetic risk for T2D and adhered to a favorable lifestyle, the HR of those having very high genetic risk and unfavorable lifestyle was 2.69 (95% CI 2.05–3.52). In addition, participants at the highest expected risk—those having very high genetic risk for T2D, non-favorable lifestyle, and poor metabolic health—exhibited substantially increased CVD risk, at 6.5 times higher than that of participants having the lowest expected risk (low T2D PRS, favorable lifestyle, and no component of metabolic syndrome) (Additional file 1: Table S9, *P* for trend < 0.001).

**Fig. 3:**
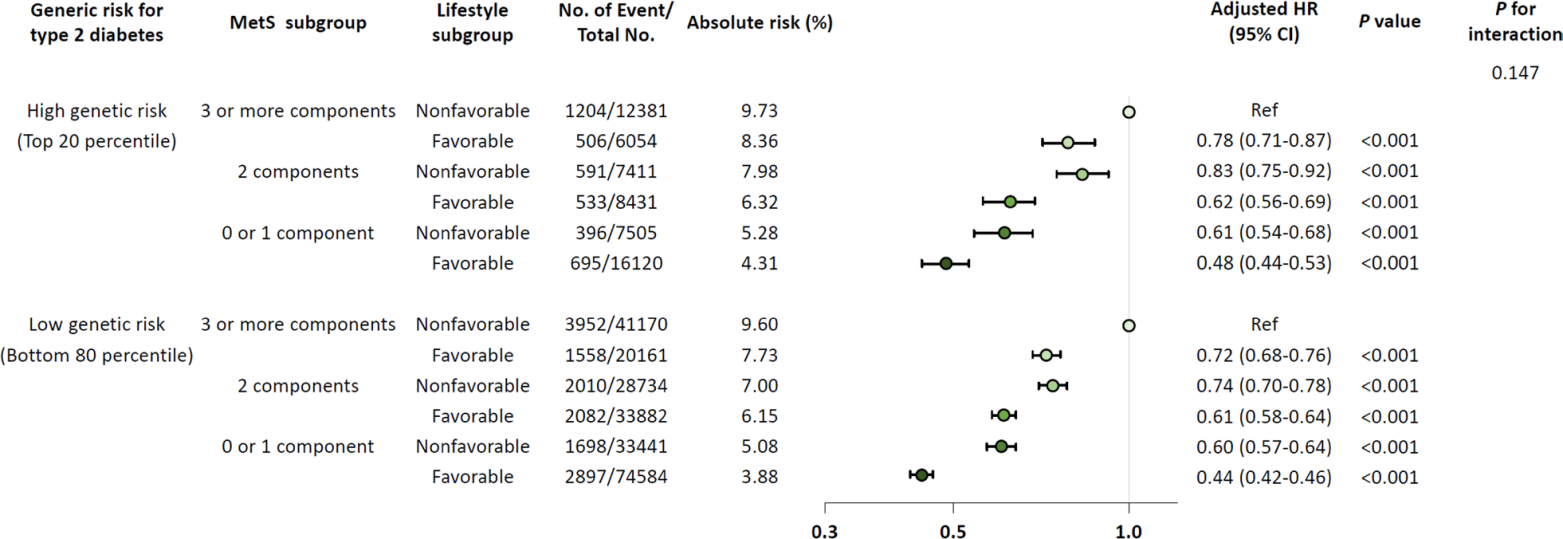
Forest plot for cardiovascular disease risk reduced by metabolic health status and lifestyle behavior in high and low genetic risk group for type 2 diabetes. Participants were categorized according to type 2 diabetes genetic risk into the following two subgroups: low (0–80th percentile), high (80–99th percentile). MetS, metabolic syndrome; HR, hazard ratio; CI, confidence interval

Overall, adherence to a favorable lifestyle reduced risk of CVD by 47% (Additional file 1: Table S10). Subgroup analyses revealed this risk reduction to occur regardless of genetic risk group; that is, metabolic health and a favorable lifestyle reduced subsequent risk of CVD by 52% among those with high genetic risk and by 56% in those with low genetic risk (Fig. 3, *P* for interaction = 0.147). Even for participants in the top 20 percentile for genetic risk of T2D who also had three or more components of metabolic syndrome, adherence to a favorable lifestyle significantly reduced risk for CVD by 22%. Sensitivity analyses which used different risk categories for low/high genetic risk did not differ in terms of the main associations (Additional file 1: Tables S13–14). Notably, the risk reduction conferred by lifestyle behaviors or metabolically healthy status was higher in the young age group (Additional file 1: Figs. S5–6). The population attributable fraction (PAF) of lifestyle modification from non-favorable to favorable was 17.2% (95% CI 16.0–18.4%), and that of metabolic health (two or more components to zero or one) was 31.1% (95% CI 29.7–32.5%). Both PAFs were greater in the young age group than in the elderly (Additional file 1: Table S15).

## Discussion

In this prospective cohort study, we comprehensively investigated the association between T2D genetic risk, lifestyle, metabolic health, and subsequent risk for CVD in middle-aged participants without prior history of CVD. Our results showed that compared with those at low genetic risk, participants at very high genetic risk for T2D, equivalent to the top 1% of the total population, had 35% higher risk of subsequent overall CVD during the nine-year follow-up period. T2D PRS was also significantly associated with multiple CVD subtypes, such as PAD, HF, atrial fibrillation/flutter, and CV mortality. Adherence to a favorable lifestyle and metabolically healthy status had the effect of reducing risk for CVD events regardless of T2D genetic risk. These risk reductions were stronger in the young age group than in the old age group.

Previous studies have investigated shared genetic components and the causal relationship between T2D and CVD. Several recent GWASs have identified polymorphisms in insulin receptor substrate 1 (*IRS1*) for which the diabetogenic allele is associated with increased coronary heart disease (CHD) risk at genome-wide significance [7, 27, 28]. Genetic variants associated with T2D risk factors such as glycemic variability or polymorphisms in angiotensin converting enzyme 2 (*ACE2*) have shown pleiotropic effects, including increased risk of CVD [29]. In addition, several MR studies have confirmed the causal relationship of T2D or its risk factors with coronary heart disease, myocardial infarction. Stephanie et al. demonstrated SNPs associated with HbA1C and diabetes to also be associated with increased risk of CAD [9]. Ahmad et al. suggested that in individuals without diabetes, genetic variants for fasting glucose are associated with increased risk of CHD [30]. Zhao et al. found that individuals having 16 variants associated with T2D also have significantly higher risk for ease [8]. Likewise, an MR study from the China Kadoorie Biobank identified causality of T2D for CHD using 48 T2D-associated SNPs [10]. In line with previous studies, our results demonstrated that genetic risk of T2D, calculated as the weighted sum of more than 6 million common variants and their respective associations with T2D, is significantly associated with increased subsequent risk of overall CVD. This association was not attenuated after adjustment for socioeconomic factors and major comorbidities that could cause confounding effects. However, adjustment for glycemic status, lipid profiles, renal function, and inflammatory markers did attenuate the association between T2D PRS and CVD, indicating the possibility of a causal pathway by which the genetic effect of T2D on CVD risk can be mediated. In our findings, adjustment for CAD PRS did not influence the main association. Although a previous report suggested the existence of a positive genetic correlation between T2D and CAD, identified using Linkage disequilibrium score regression, there was no significant correlation between T2D PRS and CAD PRS in our study [31]. Several studies have demonstrated that an integrative PRS representing multiple markers or diseases can be more helpful in disease prediction than a PRS developed for a single marker or disease. Therefore, we can expect that applying T2D PRS for stratification of CVD risk can additionally screen individuals at high risk of CVD that cannot be detected using CAD PRS alone.

A previous report indicated predisposition to CHD to be strongly associated with atherosclerotic burden in individuals with T2D, independent of traditional clinical risk factors [32]. We found that not only the risk of atherosclerotic CVD (ASCVD) subtypes such as CAD and PAD but also that of non-ASCVD subtypes including HF and atrial fibrillation/flutter was significantly higher in individuals with higher genetic risk for T2D. To the best of our knowledge, no report has yet indicated that genetic risk for T2D relates to non-ASCVD outcomes such as HF or arrhythmia; moreover, few studies have examined genetic risk of T2D and its relation to CV mortality. The underlying pathophysiology of increased risk for T2D can be attributed to structural, electrical/electromechanical, and autonomic changes [33], which may also have CV implications. One case–control study reported significant association of a genetic risk score for T2D with multi-vessel disease and severe CAD [34]; another reported T2D-associated variant to show borderline significant or insignificant association with mortality [35]. Both of these studies enrolled a relatively small number of subjects, used only tens or hundreds of SNPs, and featured relatively short follow-up periods. A recent study demonstrated diabetes to be associated with worse long-term outcomes in young adults after early-onset myocardial infarction. Given the aggressive nature of premature CVD and the impact of T2D PRS on age of CVD onset, high genetic predisposition for T2D may adversely affect prognosis for CVD [36]. Our findings indicate that T2D PRS is not associated with ischemic or hemorrhagic stroke. Unlike the consistent reports regarding association of T2D-related genetic risk with CHD, conflicting results have been reported on the association of genetic risk for T2D or T2D-related markers with stroke outcome [37, 38]. Further studies are needed to clarify this relationship.

While several studies have separately considered association of cardiometabolic abnormalities with genetic risk, lifestyle habits [39, 40], and metabolic profiles [12], none to date have systematically approached the relationship of lifestyle and metabolic health factors with genetic risk for T2D. In our study, individuals who had high genetic predisposition to T2D, adhered to a poor lifestyle, and were metabolically unhealthy had over sixfold increased risk of subsequent CVD events. Our results present a graded increment of risk according to the combination of genetic, lifestyle, and metabolic health factors. When individuals adhered to a favorable lifestyle and maintained metabolically healthy status, they evidenced no significantly increase in CVD risk regardless of genetic risk for T2D. However, even among individuals practicing a favorable lifestyle, having one or more adverse metabolic profile components was associated with significant increased risk of CVD; this relation was also irrespective of genetic risk for T2D. Previous studies have shown that lifestyle influences cardiometabolic phenotype beyond the effect of polygenic risk [41]. These findings support that additional reduction of CVD risk can be achieved through intensive treatment for metabolic health with lifestyle modification in both high and low genetic risk groups. As the risk reduction effects were more prominent in the young age group, early screening of genetic risk and intensive intervention regarding lifestyle and metabolic factors in high-risk young individuals could be an effective strategy for maximizing CVD risk reduction.

Although clinical risk prediction models are among the strategies suggested for CVD prevention, these models frequently do not provide sufficient precision at the individual level [42]. PRSs have considerable promise as screening methods for preventive medicine [43]. Using a PRS, we can obtain information predictive of genetic risk early in life, and can provide risk stratification on the basis of only a single measurement. Consequently, individuals at high risk for CV events can be provided with opportunities for early targeted prevention prior to the manifestation of clinical factors [44]. In the present work, we confirmed that genetic predisposition to T2D significantly contributes to CVD risk and interacts with lifestyle and metabolic health in relation to CVD events. Numerous studies have been published on the beneficial effects of multidisciplinary intervention by healthcare providers to promote favorable lifestyles and metabolic health [45]. However, due to limited medical resources, intensive multidisciplinary interventions to improve CV outcomes cannot be provided to all individuals. Previous studies have constantly demonstrated that beginning intervention very early can be effective in preventing chronic disease over the life course [46–48]. Using PRSs, lifestyles and metabolic health can be optimized from infancy, and genetic information can potentially influence motivation for disease prevention from very early in a patient’s life [49]. In our study, individuals with high T2D PRS tended to exhibit poor lifestyle behavior and metabolic health compared to those with lower T2D PRS, and risk reduction was more prominent in younger adults, suggesting a promising possibility that early-life intervention among those at high genetic risk of T2D will bring additional CV benefits. However, PRS-based risk estimation has been suggested to have some limitations in its application to clinical action, including the merely modest contribution of PRS to disease risk and imprecision of risk estimates [50]. In addition, there are as of yet no intervention studies or clinical trials demonstrating evidence for improved outcomes following PRS-based risk assessment. It remains necessary to validate in future studies the clinical utility and cost-effectiveness of early-life interventions guided by new prevention strategies based on genomic analyses.

## Limitations

The strengths of this study include an overall large sample and its comprehensive evaluation of genetic, lifestyle, and clinical factors, which allowed multifactorial stratification of CVD risk. Another major strength is its prospective design.

Several limitations of our study should also be considered. First, since UK Biobank participants consist of relatively healthy individuals with high socioeconomic class, they may not be fully representative of the general UK population. However, given the characteristics of the study population, the contributions of genetic risk, lifestyle, and metabolic parameters quantified here may be underestimated compared to the general population. Therefore, the potential benefit of adherence to a favorable lifestyle or metabolic health may in fact be greater. Second, the study population was restricted to participants of European ancestry aged 40 to 69 at baseline, and this association has not been validated in an independently ascertained population. Further research is warranted to investigate the degree to which these findings can be generalized to other populations. Third, lifestyle and metabolic health factors were assessed at a single time point, which did not take into account changes before or after assessment. Fourth, information regarding lifestyle behaviors was based on self-reported measures while the definition of CV outcomes was based on clinical diagnostic codes, both of which could lead to misclassification bias. Fifth, there is the possibility of unmeasured confounding factors and reverse causation bias for the relationship between genetic, lifestyle, and metabolic factors.

## Conclusions

In summary, our findings indicate that genetic risk for T2D is associated with risk of overall CVD, of diverse CVD subtypes, and of fatal CVD. In addition, adherence to a healthy lifestyle or maintaining metabolically healthy status is associated with lower risk of subsequent CVD events even in those having high genetic risk for T2D. These risk reductions were most prominent in younger participants (50 years or younger). These findings support the potential beneficial effect of using T2D PRS to triage intensive interventions to optimize personalized prevention and improve CV health. Further studies are needed to clarify the impact of an intensive intervention and the cost-effectiveness of this approach using routine genetic testing.

## Supplementary Information

**Additional file 1**. Additional figures and Tables.

## Data Availability

The UK Biobank dataset was obtained from the UK Biobank (Application Number 67855), and a full list of the variables are available online. Data cannot be shared publicly due to the violation of patient privacy and the absence of informed consent for data sharing.

## Abbreviations

ACE2: Angiotensin converting enzyme 2
AHA: American heart association
ASCVD: Atherosclerotic cardiovascular disease
BMI: Body-mass index
CAD: Coronary artrey disease
CARDIoGRAMplusC4D: Coronary artery disease genome wide replication and meta-analysis plus the coronary artery disease genetics
CCI: Charlson comorbidity index
CHD: Coronary heart disease
CI: Confidence interval
CV: Cardiovascular
CVD: Cardiovascular disease
DIAGRAM: Diabetes genetics replication and meta-analysis
GWAS: Genome-wide association study
HDL: High-density lipoprotein
HF: Heart failure
HR: Hazard ratio
IDF: International diabetes federation
IRS1: Insulin receptor substrate 1
LDL: Low-density lipoprotein
MetS: Metabolic syndrome
MR: Mendelian randomization
PAD: Peripheral artery disease
PAF: Population attributable fraction
QC: Quality control
PC: Principal component
PRS: Polygenic risk score
SD: Standard deviation
SNPs: Single-nucleotide polymorphisms
T2D: Type 2 diabetes
