# Supplementary material for: Polygenic risk for type 2 diabetes, lifestyle, metabolic health, and cardiovascular disease: a prospective UK Biobank study
Source: Cardiovasc Diabetol. 2022 Jul 14;21:131. doi: 10.1186/s12933-022-01560-2 (PMC9284808; doi:10.1186/s12933-022-01560-2)
Supplement: Supplementary file 1 — Additional file 1. Additional figures and Tables. [file 12933_2022_1560_MOESM1_ESM.pdf]

Supplemental Materials

Polygenic risk for type 2 diabetes, lifestyle, metabolic health, and cardiovascular disease: a prospective UK Biobank study

Contents

Supplemental Table 1. Detailed definitions of baseline major comorbidities and outcomes.

Supplemental Table 2. Detailed definitions of lifestyle factors, lifestyle behavior and metabolic health.

Supplemental Table 3. Number of cases with missing data for each variable.

Supplemental Table 4. Hazard ratios and 95% confidential intervals for the cardiovascular disease using Cox proportional regression model.

Supplemental Table 5. Hazard ratios and 95% confidential intervals for the cardiovascular disease using Cox proportional regression model.

Supplemental Table 6. Participant distribution according to the risk group of type 2 diabetes and coronary artery disease polygenic risk scores.

Supplemental Table 7. Study outcome summarization.

Supplemental Table 8. Hazard ratios and 95% confidential intervals for the cardiovascular mortality using Cox proportional regression model.

Supplemental Table 9. Hazard ratios and 95% confidential intervals for increased cardiovascular disease risk according to genetic risk for type 2 diabetes, metabolic health status, and lifestyle behavior.

Supplemental Table 10. Hazard ratios and 95% confidential intervals for the cardiovascular disease according to lifestyle behavior.

Supplemental Table 11. Hazard ratios and 95% confidential intervals for the cardiovascular disease according to T2D genetic risk and lifestyle behavior.

Supplemental Table 12. Hazard ratios and 95% confidential intervals for the cardiovascular disease according to T2D genetic risk and metabolic health status.

Supplemental Table 13. Sensitivity analysis of the interaction between high/low genetic risk (top 10 percentile vs. bottom 90 percentile), metabolic health, and lifestyle.

Supplemental Table 14. Sensitivity analysis of the interaction between high/low genetic risk (top 30 percentile vs. bottom 70 percentile), metabolic health, and lifestyle.

Supplemental Table 15. Papulation attributable fraction of lifestyle modification and metabolic health for cardiovascular disease.

Supplemental Figure 1. Density and prevalence plot according to genetic risk for type 2 diabetes distribution.

Supplemental Figure 2. Prevalence plot for CVD according to the Quantile groups for genetic risk for type 2 diabetes.

Supplemental Figure 3. Scatterplot of the relationship between type 2 diabetes and coronary artery disease polygenic risk scores.

Supplemental Figure 4. Forest plot for cardiovascular disease according to genetic risk for type 2 diabetes and lifestyle behavior.

Supplemental Figure 5. Forest plot for cardiovascular disease of age interaction according to genetic risk for type 2 diabetes and lifestyle behavior using reference group as unfavorable lifestyle.

Supplemental Figure 6. Forest plot for cardiovascular disease of age interaction according to genetic risk for type 2 diabetes and metabolic health status using reference group as metabolically unhealthy subgroup.

Supplemental Table 1. Detailed definitions of baseline major comorbidities and outcomes.

Disease	Path	Field ID	Code
Baseline dyslipidemia	Verbal interview	Non-cancer illness, self-report (20002)	High cholesterol (1473)
	First occurrence before enrollment	First reported of disorders of lipoprotein metabolism and other lipidemia (130815, 130816)	E78.x
	Medication	Medication for cholesterol, blood pressure or diabetes (6177)	Cholesterol lowering medication
Baseline hypertension	Verbal interview	Non-cancer illness, self-report (20002)	1065, 1072
	Touchscreen	Vascular/heart problems diagnosed by doctor (6150)	High blood pressure
	First occurrence before enrollment	First reported of essential hypertension (131286, 131287)	I10.x
		First reported of hypertensive heart disease (131288, 131289)	I11.x
		First reported of hypertensive renal disease (131290, 131291)	I12.x
		First reported of hypertensive heart and renal disease (131292, 131293)	I13.x
		First reported of secondary hypertension (131294, 131295)	I15.x
	Medication	Medication for cholesterol, blood pressure or diabetes (6177)	Blood pressure medication
Baseline type 2 diabetes mellitus	Verbal interview	Non-cancer illness, self-report (20002)	Diabetes (1220)<br>Type 2 diabetes (1223)
	Touchscreen	Diabetes diagnosed by doctor (2443)	Yes
	First occurrence before enrollment	First reported of non-insulin-dependent diabetes mellitus (130708, 130709)	E11.x
		First reported of unspecified diabetes mellitus (130714, 130715)	E14.x
	Medication	Treatment/medication code (20003)	Insulin (1140883066)<br>Metformin (1140884600, 1141189090)<br>Sulfonylurea (1141152590, 1140874744, 1140874718, 1141156984)<br>Acarbose (1140868902)<br>Thiazolidinedione (1141171646)<br>Meglitinide (1141168660, 1141173882)
	HbA1c at baseline	Glycated hemoglobin (HbA1c) (30750)	≥6.5%
	Verbal interview for exclusion type 1 diabetes	Non-cancer illness, self-report (20002)	Type 1 diabetes (1222)
	First occurrence for exclusion type 1 diabetes	First reported of insulin-dependent diabetes mellitus (130706, 130707)	E10.x
Baseline coronary artery disease	Verbal interview	Non-cancer illness, self-report (20002)	1074 (angina)<br>1075 (heart attack/myocardial infarction)
	Touchscreen	Vascular/heart problems diagnosed by doctor (6150)	Heart attack, angina
	First occurrence before enrollment	First reported of angina pectoris (131296, 131297)	I20.x
		First reported of acute myocardial infarction (131298, 131299)	I21.x

		First reported of subsequent myocardial infarction (131300, 131301)	I22.x
		First reported of certain current complications following acute myocardial infarction (131302, 131303)	I23.x
		First reported of other acute ischemic heart diseases (131304, 131305)	I24.x
		First reported of chronic ischemic heart disease (131306, 131307)	I25.x
Baseline peripheral artery disease	Verbal interview	Non-cancer illness, self-report (20002)	Peripheral vascular disease (1067)
	Hospital inpatient data	Summary Diagnosis (ICD10, 41270)	I70.0, I70.00, I70.01, I70.2, I70.21, I70.8, I70.80, I70.9, I70.90, I73.8, I73.9
		Summary Diagnosis (ICD9, 41271)	4400, 4402, 4438, 4439
Baseline heart failure	Verbal interview	Non-cancer illness, self-report (20002)	Heart failure (1076, 1079)
	Hospital inpatient data	Summary Diagnosis (ICD10, 41270)	I50, I50.0, I50.1, I50.9
		Summary Diagnosis (ICD9, 41271)	4254, 4280, 4281, 4289
Baseline heart arrhythmia	Verbal interview	Non-cancer illness, self-report (20002)	Heart arrhythmia (1094)
	First occurrence before enrollment	First reported of atrial fibrillation/flutter (131350, 131351)	I48.x
		First reported of other cardiac arrhythmias (131352, 131353)	I49.x
Baseline ischemic stroke	Verbal interview	Non-cancer illness, self-report (20002)	Stroke (1081,1082,1583)
	First occurrence before enrollment	First reported of cerebral infarction (131366, 131367)	I63.x
Baseline hemorrhagic stroke	Verbal interview	Non-cancer illness, self-report (20002)	Stroke (1081)
	First occurrence before enrollment	First reported of subarachnoid hemorrhage (131360, 131361)	I60.x
		First reported of intracerebral hemorrhage (131362, 131363)	I61.x
		First reported of other nontraumatic intracranial hemorrhage (131364, 131365)	I62.x
Baseline chronic lung disease	Verbal interview		Asthma (1111)
		Non-cancer illness, self-report (20002)	Chronic obstructive airway disease/COPD (1112)<br>Emphysema/chronic bronchitis (1113)<br>Bronchiectasis (1114)<br>Interstitial lung disease (1115)
	First occurrence before enrollment	First reported of bronchitis, not specified as acute or chronic (131484, 131485)	J40.x
		First reported of simple and mucopurulent chronic bronchitis (131486, 131487)	J41.x
		First reported of unspecified chronic bronchitis (131488, 131489)	J42.x
		First reported of emphysema (131490, 131491)	J43.x
		First reported of other chronic obstructive pulmonary disease (131492, 131493)	J44.x
		First reported of asthma (131494, 131495)	J45.x
		First reported of status asthmaticus (131496, 131497)	J46.x

		First reported of bronchiectasis (131498, 131499)	J47.x
		First reported of coalworker's pneumoconiosis (131500, 131501)	J60.x
		First reported of pneumoconiosis due to asbestos and other mineral fibers (131502, 131503)	J61.x
		First reported of pneumoconiosis due to dust containing silica (131504, 131505)	J62.x
		First reported of pneumoconiosis due to other inorganic dusts (131506, 131507)	J63.x
		First reported of unspecified pneumoconiosis (131508, 131509)	J64.x
		First reported of airway disease due to specific organic dust (131512, 131513)	J66.x
		First reported of hypersensitivity pneumonitis due to organic dust (131514, 131515)	J67.x
Baseline chronic liver disease	Verbal interview		Hepatitis (1155)
		Non-cancer illness, self-report (20002)	Infective/viral hepatitis (1156)<br>Non-infective hepatitis (1157)<br>Liver failure/cirrhosis (1158)
	First occurrence before enrollment	First reported of chronic viral hepatitis (130200, 130201)	B18.x
		First reported of esophageal varices (131406, 131407)	I85.x
		First reported of alcoholic liver disease (131658, 131659)	K70.x
		First reported of toxic liver disease (131660, 131661)	K71.x
		First reported of hepatic failure, not elsewhere classified (131662, 131663)	K72.x
		First reported of chronic hepatitis, not elsewhere classified (131664, 131665)	K73.x
		First reported of other diseases of liver (131670, 131671)	K76.x
Baseline chronic kidney disease	Verbal interview		Renal/kidney failure (1192)
		Non-cancer illness, self-report (20002)	Renal failure requiring dialysis (1193)<br>Renal failure not requiring dialysis (1194)
	First occurrence before enrollment	First reported of chronic renal failure (132030, 132031)	N18.x
		First reported of unspecified renal failure (132032, 132033)	N19.x
Baseline cancer	Verbal interview	Cancer, self-report (20001)	1001-1012, 1015-1048, 1050-1053, 1055,1056, 1058-1068, 1070-1082, 1084-1088
	First occurrence before enrollment	Cancer register (40006)	C00-C97
Coronary artery disease outcome	First occurrence before enrollment	First reported of angina pectoris (131296, 131297)	I20.x
		First reported of acute myocardial infarction (131298, 131299)	I21.x
		First reported of subsequent myocardial infarction (131300, 131301)	I22.x

		First reported of certain current complications following acute myocardial infarction (131302, 131303)	I23.x
		First reported of other acute ischemic heart diseases (131304, 131305)	I24.x
		First reported of chronic ischemic heart disease (131306, 131307)	I25.x
Peripheral artery disease outcome	Hospital inpatient data	Summary Diagnosis (ICD10, 41270)	I70.0, I70.00, I70.01, I70.2, I70.21, I70.8, I70.80, I70.9, I70.90, I73.8, I73.9
		Summary Diagnosis (ICD9, 41271)	4400, 4402, 4438, 4439
Heart failure outcome	Hospital inpatient data	Summary Diagnosis (ICD10, 41270)	I50, I50.0, I50.1, I50.9
		Summary Diagnosis (ICD9, 41271)	4254, 4280, 4281, 4289
Atrial fibrillation/atrial flutter outcome	First occurrence before enrollment	First reported of atrial fibrillation/flutter (131350, 131351)	I48.x
Ischemic stroke	First occurrence before enrollment	First reported of cerebral infarction (131366, 131367)	I63.x
Hemorrhagic stroke	First occurrence before enrollment	First reported of subarachnoid hemorrhage (131360, 131361)	I60.x
		First reported of intracerebral hemorrhage (131362, 131363)	I61.x
		First reported of other nontraumatic intracranial hemorrhage (131364, 131365)	I62.x

Supplemental Table 2. Detailed definitions of lifestyle factors, lifestyle behavior and metabolic health.

**Lifestyle factors**	**Component**	**Healthy lifestyle or metabolic status**	**Field ID of UK biobank**
Current smoking	Current smoking at baseline	Absence	20116
Obesity	BMI at baseline	<30 kg/m^2	21001
Physical activity	Number of days per week of physical activity 10+ minutes	Participating in moderate activity ≥5 days a week or vigorous activity ≥3 days a week	884 (Moderate physical activity 10+ minutes)<br>904 (Vigorous physical activity 10+ minutes)
Eating habits	At least half of all following diet components was considered as a healthy lifestyle, less than half was considered as an unhealthy lifestyle		
	Fruit	≥3 serving/day	1309 (Fresh fruit)<br>1319 (Dried fruit)
	Vegetable	≥3 serving/day	1289 (Cooked vegetables)<br>1299 (Salad or raw vegetables)
	Whole grains	≥3 serving/day	1438, 1448 (Wholemeal or wholegrain bread)<br>1458, 1468 (Bran, oat, muesli cereal)
	Fish	≥2 serving/week	1329 (Oily fish)<br>1339 (Non-oily fish)
	Dairy	≥2.5 serving/week	1408 (Cheese)<br>1418 (Milk)
	Refined grains	≤1.5 serving/week	1438, 1448 (Wholemeal or wholegrain bread)<br>1458, 1468 (Bran, oat, muesli cereal)
	Processed meats	≤1 serving/week	1349 (Processed meat)<br>3680 (Age when last ate any kind of meat, 0 if indicated having never eaten meat)
	Unprocessed meats	≤1.5 serving/week	1359 (Poultry)<br>1369 (Beef)<br>1379 (Lamb)<br>1389 (Pork)<br>3680 (Age when last ate any kind of meat, 0 if indicated having never eaten meat)
	Sugar-sweetened beverages	≤1 serving/week	6144 (Never eats sugar or foods/drinks containing sugar)
Lifestyle behavior	Favorable	Having at least three healthy lifestyle factors	
	Intermediate	Having two healthy lifestyle factors	
	Unfavorable	Having one or fewer healthy lifestyle factor	
Components of metabolic syndrome	Waist circumference	Men <102 cm<br>Women <88 cm	48
	Triglyceride	<1.70 mmol/L	30870
	HDL cholesterol	≥1.03 mmol/L	30760
	Glucose<br>(or having diabetes)	≥5.6 mmol/L	30740
	Systolic blood pressure<br>or diastolic blood pressure	≥130 mmHg or<br>≥ 85 mmHg	4080<br>4079

Supplemental Table 3. Number of cases with missing data for each variable.

Variable	n (%)
Total	345217
Systolic blood pressure	294 (0.1)
Diastolic blood pressure	292 (0.1)
Height	648 (0.2)
Weight	844 (0.3)
Body mass index	942 (0.3)
Waist circumference	521 (0.2)
Lifestyle behavior	12550 (3.6)
Total cholesterol	16011 (4.6)
Triglyceride	16274 (4.7)
HDL-cholesterol	43959 (12.7)
LDL-cholesterol	16626 (4.8)
Creatinine	16190 (4.7)
HbA1c	16237 (4.7)
Income	47173 (13.7)
Townsend deprivation index	415 (0.1)

Supplemental Table 4. Hazard ratios and 95% confidential intervals for the cardiovascular disease using Cox proportional regression model.

		No. of Events/ Total No.	Incidence rate per 1000 person-yr (95% CI)	Absolute risk (%)	Crude		Model 1		Model 2		Model 3	
					HR (95% CI)	*P* value	HR (95% CI)	*P* value	HR (95% CI)	*P* value	HR (95% CI)	*P* value
T2D PRS	**Low risk**	4045/70210	6.74 (6.53-6.95)	5.86	Ref		Ref		Ref		Ref	
	**Intermediate risk**	13115/210634	7.30 (7.28-7.43)	6.33	1.08 (1.05-1.12)	<0.001	1.10 (1.06-1.14)	<0.001	1.09 (1.05-1.13)	<0.001	1.09 (1.05-1.13)	<0.001
	**High risk**	4443/66608	7.84 (7.62-8.08)	6.78	1.16 (1.12-1.21)	<0.001	1.19 (1.14-1.24)	<0.001	1.17 (1.11-1.22)	<0.001	1.16 (1.11-1.21)	<0.001
	**Very high risk**	262/3510	8.81 (7.78-9.94)	7.59	1.30 (1.15-1.48)	<0.001	1.35 (1.19-1.53)	<0.001	1.27 (1.10-1.46)	<0.001	1.30 (1.15-1.48)	<0.001
***P* for trend**						<0.001		<0.001				<0.001

Model 1 was adjusted for age, sex, genotyping array, first ten principal components.

Model 2 was adjusted for model 1 components, income level, and Townsend deprivation index.

Model 3 was adjusted for model 1 components, and CAD PRS.

Abbreviations: T2D, type 2 diabetes; CAD, coronary artery disease; PRS, polygenic risk score; HR, hazard ratio; CI, confidence interval.

Supplemental Table 5. Hazard ratios and 95% confidential intervals for the cardiovascular disease using Cox proportional regression model.

		No. of Events/ Total No.	Incidence rate per 1000 person-yr (95% CI)	Absolute risk (%)	Model 1		Model 2		Model 3	
					**HR (95% CI)**	***P* value**	**HR (95% CI)**	***P* value**	**HR (95% CI)**	***P* value**
**T2D PRS**	**Low risk**	4045/70210	6.74 (6.53-6.95)	5.86	Ref		Ref		Ref	
	**Intermediate risk**	13115/210634	7.30 (7.28-7.43)	6.33	1.07 (1.03-1.11)	0.002	1.06 (1.02-1.10)	0.002	1.10 (1.06-1.13)	<0.001
	**High risk**	4443/66608	7.84 (7.62-8.08)	6.78	1.13 (1.08-1.19)	<0.001	1.11 (1.06-1.16)	<0.001	1.18 (1.13-1.23)	<0.001
	**Very high risk**	262/3510	8.81 (7.78-9.94)	7.59	1.20 (1.03-1.39)	0.019	1.17 (1.02-1.35)	0.022	1.33 (1.18-1.51)	<0.001
***P* for trend**						<0.001		<0.001		<0.001

Model 1 was adjusted for age, sex, genotyping array, first ten principal components, BMI, current smoking, alcohol frequency, physical activity, and eating habits.

Model 2 was adjusted for Model 1 components, systolic BP, diastolic BP, HbA1c, triglyceride, HDL cholesterol, LDL cholesterol, and estimated GFR.

Model 3 was adjusted for Model 1 components, cancer, chronic lung disease, chronic liver disease, and chronic kidney disease.

Abbreviations: T2D, type 2 diabetes; PRS, polygenic risk score; BP, blood pressure; eGFR, estimated glomerular filtration rate; HR, hazard ratio; CI, confidence interval.

Supplemental Table 6. Participant distribution according to the risk group of type 2 diabetes and coronary artery disease polygenic risk scores.

**LDpred*** Khera *et al.,* 2018		T2D PRS			
		Low risk 0-20th	Intermediate 21-80th	High 79-98th	Very high 99th
CAD PRS	Low risk 0-20th	15401	41613	11462	581
	Intermediate 21-79th	41277	124293	39553	1997
	High 80-98th	11817	39164	13793	814
	Very high 99th	562	2050	780	60

*The PRSs for T2D and CAD respectively derived from the DIAGRAM consortium and CARDIOGRAMplusC4D consortium, which were based on the pre-calculated weights for SNPs provided by Khera et al. 2018.

Abbreviations: T2D, type 2 diabetes; CAD, coronary artery disease; PRS, polygenic risk score.

Supplemental Table 7. Study outcome summarization.

	Total	T2D PRS				
		**Low** 0-19th percentile	**Intermediate** 20-79th percentile	**High** 80-98th percentile	**Very high** 99th percentile	***P* value**
	**(n=345217)**	**(n=69070)**	**(n=207192)**	**(n=65505)**	**(n=3450)**	
Cardiovascular events during follow-up periods	21865 (6.3)	4045 (5.9)	13115 (6.3)	4443 (6.8)	262 (7.6)	<0.001
Coronary artery disease	14307 (4.1)	2597 (3.8)	8580 (4.1)	2947 (4.5)	183 (5.3)	<0.001
Peripheral artery disease	2054 (0.6)	366 (0.5)	1198 (0.6)	468 (0.7)	22 (0.7)	<0.001
Heart failure	2954 (0.9)	528 (0.8)	1803 (0.9)	579 (0.9)	44 (1.3)	0.002
Atrial fibrillation/Atrial flutter	5677 (1.6)	1092 (1.6)	3396 (1.6)	1116 (1.7)	73 (2.1)	0.048
Ischemic stroke	2328 (0.7)	444 (0.6)	1421 (0.7)	440 (0.7)	23 (0.7)	0.696
Hemorrhagic stroke	1117 (0.3)	209 (0.3)	675 (0.3)	225 (0.3)	8 (0.2)	0.444
Cardiovascular death during follow-up periods	1963 (0.6)	351 (0.5)	1177 (0.6)	408 (0.6)	27 (0.8)	0.014

Data are n (%) or mean (SD).

Abbreviations: T2D, type 2 diabetes; PRS, polygenic risk score.

Supplemental Table 8. Hazard ratios and 95% confidential intervals for the cardiovascular mortality using Cox proportional regression model.

		Cardiovascular mortality				
		No. of Events/ Total No.	Incidence rate per 1000 person-yr (95% CI)	Absolute risk (%)	Model 1	
					HR (95% CI)	*P* value
**T2D PRS**	Low risk	351/69070	0.57 (0.51-0.63)	0.51	Ref	
	Intermediate risk	1177/207192	0.64 (0.60-0.67)	0.57	1.14 (1.01-1.29)	0.03
	High risk	408/65505	0.70 (0.63-0.77)	0.62	1.27 (1.10-1.46)	0.001
	Very high risk	27/3450	0.88 (0.58-1.28)	0.78	1.63 (1.10-2.41)	0.015

Model 1 was adjusted for age, sex, genotyping array, and first ten principal components.

Abbreviations: T2D, type 2 diabetes; PRS, polygenic risk score; HR, hazard ratio; CI, confidence interval.

Supplemental Table 9. Hazard ratios and 95% confidential intervals for increased cardiovascular disease risk according to genetic risk for type 2 diabetes, metabolic health status, and lifestyle behavior.

T2D PRS subgroup	MS subgroup	Lifestyle subgroup	No. of Events/ Total No.	Absolute risk (%)	Model 1	
					HR (95% CI)	*P* value
Low genetic risk	0 component of MetS	Favorable lifestyle	143/6685	2.14	Ref	
		Nonfavorable lifestyle	408/17519	2.33	1.07 (0.72-1.61)	0.729
	1 component of MetS	Favorable lifestyle	117/4770	2.45	0.91 (0.52-1.59)	0.734
		Nonfavorable lifestyle	3/198	1.52	2.16 (0.52-9.00)	0.292
	2 components of MetS	Favorable lifestyle	657/13382	4.91	2.34 (1.61-3.40)	<0.001
		Nonfavorable lifestyle	1689/36998	4.57	2.38 (1.66-3.40)	<0.001
	3 or more components of MetS	Favorable lifestyle	553/10653	5.19	2.86 (1.96-4.16)	<0.001
		Nonfavorable lifestyle	22/499	4.41	1.76 (0.69-4.52)	0.239
Intermediate genetic risk	0 component of MetS	Favorable lifestyle	471/8470	5.56	2.83 (1.93-4.14)	<0.001
		Nonfavorable lifestyle	1611/25412	6.34	3.51 (2.31-4.92)	<0.001
	1 component of MetS	Favorable lifestyle	503/8028	6.27	3.37 (2.31-4.92)	<0.001
		Nonfavorable lifestyle	30/403	7.44	3.61 (1.72-7.56)	<0.001
	2 components of MetS	Favorable lifestyle	348/4652	7.48	4.08 (2.77-6.01)	<0.001
		Nonfavorable lifestyle	1210/15509	7.80	4.14 (2.89-5.93)	<0.001
	3 or more components of MetS	Favorable lifestyle	470/5719	8.22	3.76 (2.56-5.51)	<0.001
		Nonfavorable lifestyle	33/335	9.85	5.48 (3.86-7.79)	<0.001
High genetic risk	0 component of MetS	Favorable lifestyle	92/2744	3.35	1.76 (1.03-3.01)	0.039
		Nonfavorable lifestyle	220/7088	3.10	1.85 (1.21-2.84)	0.005
	1 component of MetS	Favorable lifestyle	63/1952	3.23	2.09 (1.19-3.69)	0.011
		Nonfavorable lifestyle	3/89	3.37	4.76 (1.14-19.88)	0.032
	2 components of MetS	Favorable lifestyle	362/6204	5.84	3.21 (2.17-4.75)	<0.001
		Nonfavorable lifestyle	1024/17405	5.88	3.22 92.24-4.63)	<0.001
	3 or more components of MetS	Favorable lifestyle	319/5201	6.13	3.81 (2.57-5.65)	<0.001
		Nonfavorable lifestyle	11/263	4.18	3.48 (1.36-8.94)	0.009
Very high genetic risk	0 component of MetS	Favorable lifestyle	472/6938	6.80	3.83 (2.62-5.59)	<0.001
		Nonfavorable lifestyle	1538/21796	7.06	3.79 (2.65-5.42)	<0.001
	1 component of MetS	Favorable lifestyle	559/7079	7.90	4.11 (2.82-6.00)	<0.001
		Nonfavorable lifestyle	32/332	9.64	4.50 (2.15-9.43)	<0.001
	2 components of MetS	Favorable lifestyle	846/9090	9.31	4.89 (3.39-7.04)	<0.001
		Nonfavorable lifestyle	3106/32080	9.68	5.31 (3.73-7.54)	<0.001
	3 or more components of MetS	Favorable lifestyle	1125/11627	9.68	5.43 (3.79-7.79)	<0.001
		Nonfavorable lifestyle	79/754	10.48	6.54 (3.96-10.82)	<0.001

Model 1 was adjusted for age, sex, genotyping array, and first ten principal components.

Abbreviations: T2D, type 2 diabetes; PRS, polygenic risk score; MetS, metabolic syndrome; HR, hazard ratio; CI, confidence interval.

Supplemental Table 10. Hazard ratios and 95% confidential intervals for the cardiovascular disease according to lifestyle behavior.

		Cardiovascular disease			
		No. of Events/ Total No.	Absolute risk (%)	Model 1	
				HR (95% CI)	*P* value
**Lifestyle behavior**	Unfavorable lifestyle	3838/39232	9.78	Ref	
	Intermediate lifestyle	7997/113231	7.06	0.71 (0.67-0.74)	<0.001
	Favorable lifestyle	9780/185728	5.27	0.53 (0.51-0.55)	<0.001

Model 1 was adjusted for age, sex, genotyping array, and first ten principal components.

Abbreviations: HR, hazard ratio; CI, confidence interval.

Supplemental Table 11. Hazard ratios and 95% confidential intervals for the cardiovascular disease according to T2D genetic risk and lifestyle behavior.

T2D PRS subgroup	Lifestyle subgroup	Cardiovascular disease			
		No. of Events/ Total No.	Absolute risk (%)	Model 1	
				HR (95% CI)	*P* value
All participants	Unfavorable lifestyle	3643/38450	9.48	Ref	
	Intermediate lifestyle	7619/111276	6.85	0.70 (0.67-0.73)	<0.001
	Favorable lifestyle	9414/182991	5.15	0.52 (0.50-0.54)	<0.001
Very high genetic risk	Unfavorable lifestyle	54/466	11.59	Ref	
	Intermediate lifestyle	91/1183	7.69	0.65 (0.46-0.91)	0.012
	Favorable lifestyle	103/1660	6.21	0.50 (0.36-0.69)	<0.001
High genetic risk	Unfavorable lifestyle	775/8069	9.61	Ref	
	Intermediate lifestyle	1578/21512	7.34	0.74 (0.68-0.81)	<0.001
	Favorable lifestyle	1856/33562	5.53	0.55 (0.51-0.60)	<0.001
Intermediate genetic risk	Unfavorable lifestyle	2178/23110	9.42	Ref	
	Intermediate lifestyle	4564/66812	6.83	0.70 (0.68-0.74)	<0.001
	Favorable lifestyle	5625/109760	5.13	0.52 (0.49-0.54)	<0.001
Low genetic risk	Unfavorable lifestyle	636/6805	9.35	Ref	
	Intermediate lifestyle	1386/21769	6.37	0.65 (0.59-0.72)	<0.001
	Favorable lifestyle	1830/38009	4.82	0.49 (0.44-0.53)	<0.001

Model 1 was adjusted for age, sex, genotyping array, and first ten principal components.

Abbreviations: T2D, type 2 diabetes; PRS, polygenic risk score; HR, hazard ratio; CI, confidence interval.

Supplemental Table 12. Hazard ratios and 95% confidential intervals for the cardiovascular disease according to T2D genetic risk and metabolic health status.

T2D PRS subgroup	MetS subgroup	Cardiovascular disease			
		No. of Events/ Total No.	Absolute risk (%)	Model 1	
				HR (95% CI)	*P* value
All participants	3 or more components	7673/83448	9.20	Ref	
	2 components	5473/81314	6.73	0.74 (0.72-0.77)	<0.001
	0 or 1 component	5961/135345	4.40	0.55 (0.53-0.57)	<0.001
Very high genetic risk	3 or more components	121/1155	10.48	Ref	
	2 components	64/760	8.42	0.79 (0.58-1.07)	0.125
	0 or 1 component	43/1081	3.98	0.42 (0.30-0.60)	<0.001
High genetic risk	3 or more components	1694/18142	9.34	Ref	
	2 components	1104/15619	7.07	0.77 (0.71-0.83)	<0.001
	0 or 1 component	1100/23215	4.74	0.57 (0.53-0.62)	<0.001
Intermediate genetic risk	3 or more components	4608/49761	9.26	Ref	
	2 components	3315/48955	6.77	0.74 (0.71-0.78)	<0.001
	0 or 1 component	3509/81240	4.32	0.53 (0.51-0.56)	<0.001
Low genetic risk	3 or more components	1250/14390	8.69	Ref	
	2 components	990/15980	6.20	0.72 (0.66-0.78)	<0.001
	0 or 1 component	1309/29809	4.39	0.58 (0.54-0.63)	<0.001

Model 1 was adjusted for age, sex, genotyping array, and first ten principal components.

Abbreviations: T2D, type 2 diabetes; PRS, polygenic risk score; MetS, metabolic syndrome; HR, hazard ratio; CI, confidence interval.

Supplemental Table 13. Sensitivity analysis of the interaction between high/low genetic risk (top 10 percentile vs. bottom 90 percentile), metabolic health, and lifestyle.

T2D PRS subgroup	MetS subgroup	Lifestyle subgroup	No. of Events/ Total No.	Absolute risk (%)	Model 1		*P* for interaction
					HR (95% CI)	*P* value	
							0.399
High genetic risk	3 or more components	Nonfavorable lifestyle	288/2769	10.40	Ref		
(Top 10 percentile)		Favorable lifestyle	120/1295	9.27	0.81 (0.65-0.99)	0.049	
	2 components	Nonfavorable lifestyle	120/1471	8.16	0.80 (0.65-0.99)	0.042	
		Favorable lifestyle	108/1666	6.48	0.59 (0.47-0.74)	<0.001	
	0-1 component	Nonfavorable lifestyle	74/1462	5.06	0.53 (0.41-0.69)	<0.001	
		Favorable lifestyle	128/3049	4.20	0.43 (0.35-0.54)	<0.001	
Low genetic risk	3 or more components	Nonfavorable lifestyle	4868/50782	9.59	Ref		
(Bottom 90 percentile)		Favorable lifestyle	1944/24920	7.80	0.73 (0.69-0.77)	<0.001	
	2 components	Nonfavorable lifestyle	2481/34674	7.16	0.76 (0.72-0.79)	<0.001	
		Favorable lifestyle	2507/40647	6.17	0.61 (0.58-0.64)	<0.001	
	0-1 component	Nonfavorable lifestyle	2020/39484	5.12	0.60 (0.57-0.64)	<0.001	
		Favorable lifestyle	3464/87655	3.95	0.45 (0.43-0.47)	<0.001	

Model 1 was adjusted for age, sex, genotyping array, and first ten principal components.

Abbreviations: T2D, type 2 diabetes; PRS, polygenic risk score; MetS, metabolic syndrome; HR, hazard ratio; CI, confidence interval.

Supplemental Table 14. Sensitivity analysis of the interaction between high/low genetic risk (top 30 percentile vs. bottom 70 percentile), metabolic health, and lifestyle.

T2D PRS subgroup	MetS subgroup	Lifestyle subgroup	No. of Events/ Total No.	Absolute risk (%)	Model 1		*P* for interaction
					HR (95% CI)	*P* value	
							0.223
High genetic risk	3 or more components	Nonfavorable lifestyle	921/9545	9.65	Ref		
(Top 30 percentile)		Favorable lifestyle	395/4624	8.54	0.81 (0.72-0.91)	<0.001	
	2 components	Nonfavorable lifestyle	446/5614	7.94	0.84 (0.75-0.94)	0.002	
		Favorable lifestyle	399/6345	6.29	0.62 (0.56-0.70)	<0.001	
	0-1 component	Nonfavorable lifestyle	300/5655	5.31	0.61 (0.54-0.70)	<0.001	
		Favorable lifestyle	523/12071	4.33	0.49 (0.44-0.54)	<0.001	
Low genetic risk	3 or more components	Nonfavorable lifestyle	4235/44006	9.62	Ref		
(Bottom 70 percentile)		Favorable lifestyle	1669/21591	7.73	0.72 (0.68-0.76)	<0.001	
	2 components	Nonfavorable lifestyle	2155/30531	7.06	0.74 (0.71-0.78)	<0.001	
		Favorable lifestyle	2216/35968	6.16	0.61 (0.58-0.64)	<0.001	
	0-1 component	Nonfavorable lifestyle	1794/35291	5.08	0.60 (0.57-0.63)	<0.001	
		Favorable lifestyle	3069/78633	3.90	0.44 (0.42-0.47)	<0.001	

Model 1 was adjusted for age, sex, genotyping array, and first ten principal components.

Abbreviations: T2D, type 2 diabetes; PRS, polygenic risk score; MetS, metabolic syndrome; HR, hazard ratio; CI, confidence interval.

Supplemental Table 15. Papulation attributable fraction of lifestyle modification and metabolic health for cardiovascular disease.

Lifestyle	Nonfavorable to favorable	All participants	17.2% (16.0 to 18.4%)
		40 to 50 years	27.1% (23.4 to 30.6%)
		51 to 69 years	15.9% (14.6 to 17.1%)
Metabolic syndrome	MetS 2 or 3 to MetS 0 or 1	All participants	31.1% (29.7 to 32.5%)
		40 to 50 years	34.5% (30.8 to 38.0%)
		51 to 69 years	26.1% (24.5 to 27.7%)

Abbreviation: MetS, metabolic syndrome.

Supplemental Figure 1. Density and prevalence plot according to genetic risk for type 2 diabetes distribution.

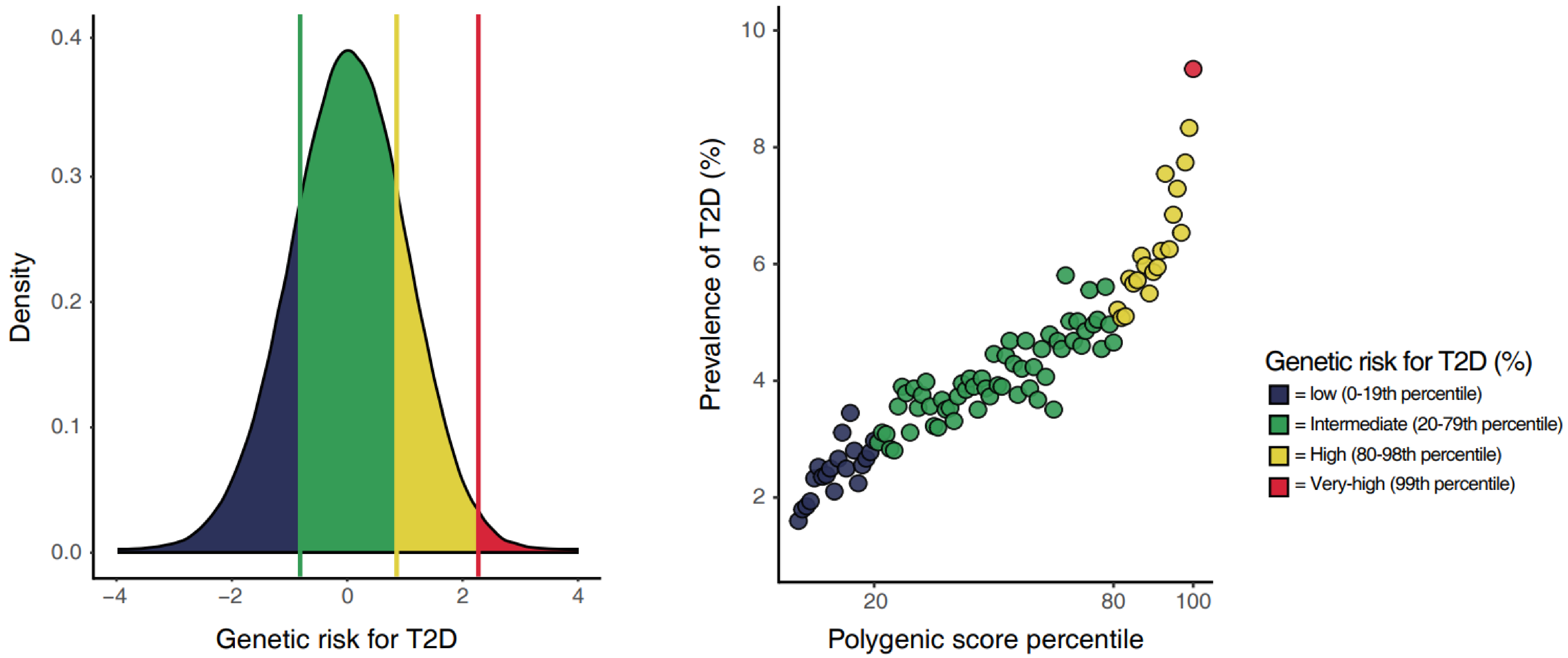

T2D PRS risk groups: low (0–19th percentile), intermediate (20–79th percentile), high (80–98th percentile), and very high (99th percentile).

Abbreviations: T2D, type 2 diabetes; PRS, polygenic risk score.

Supplemental Figure 2. Prevalence plot for CVD according to the Quantile groups for genetic risk for type 2 diabetes.

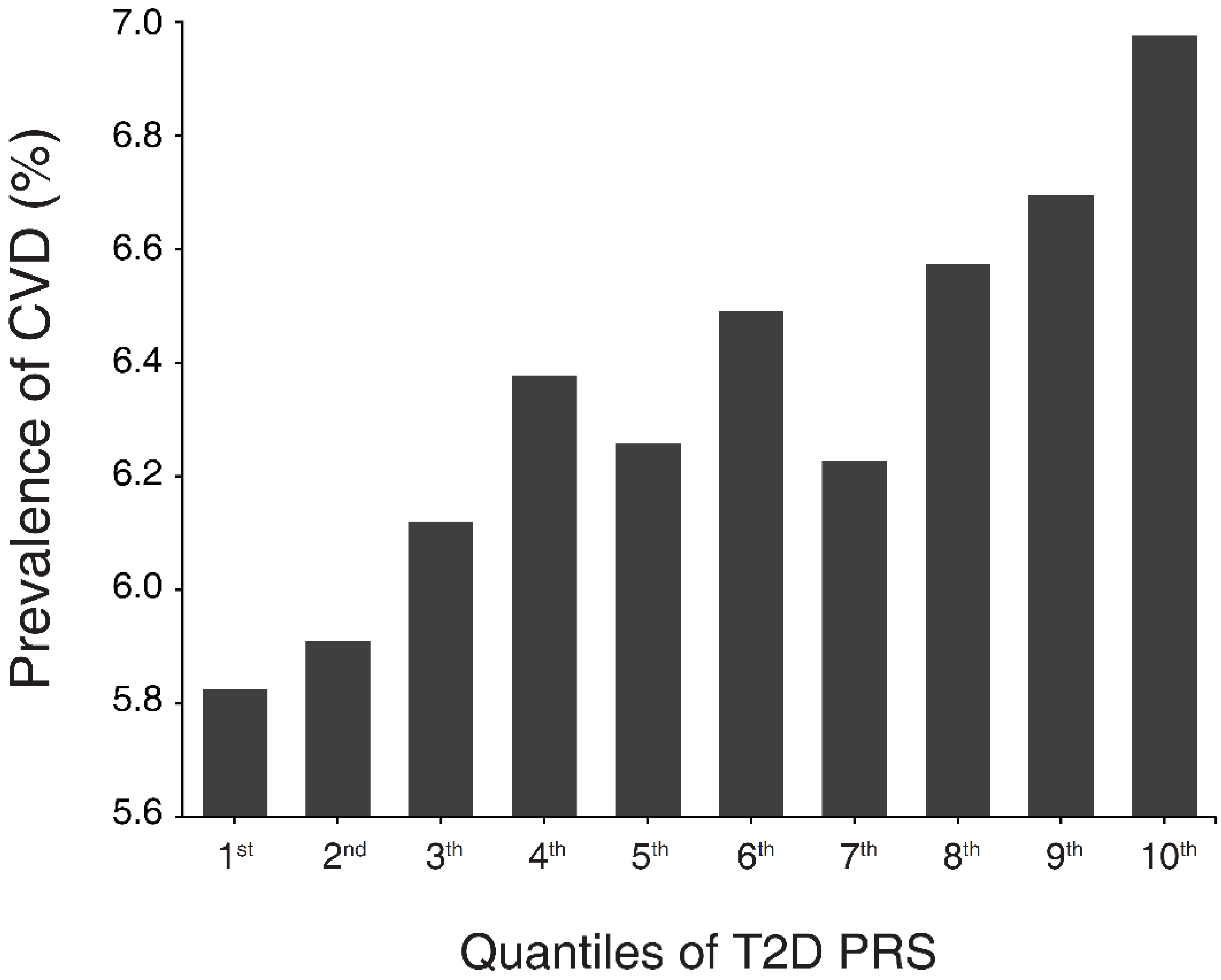

Abbreviations: CVD, cardiovascular disease; T2D, type 2 diabetes; PRS, polygenic risk score.

Supplemental Figure 3. Scatterplot of the relationship between type 2 diabetes and coronary artery disease polygenic risk scores.

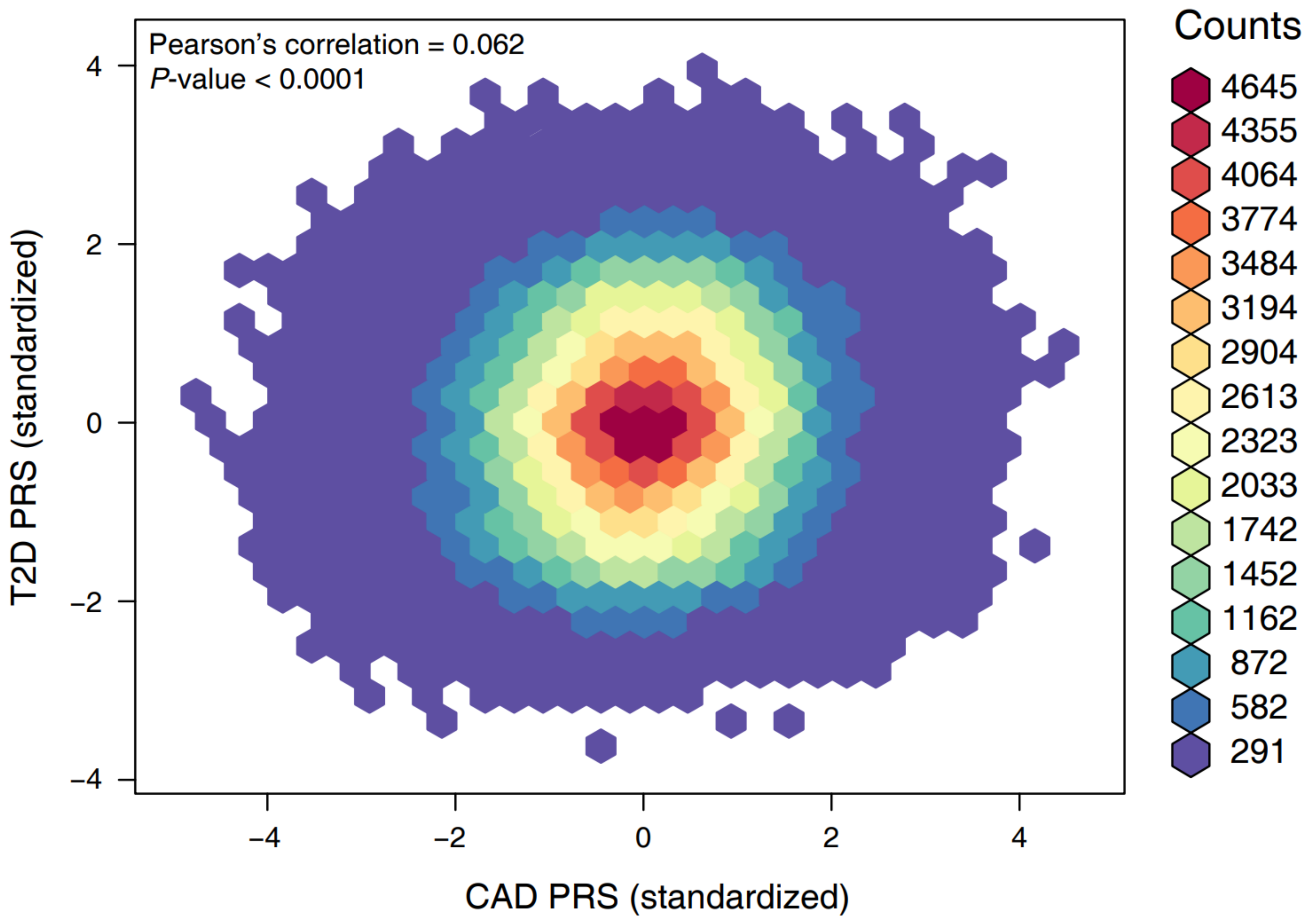

Abbreviations: T2D, type 2 diabetes; CAD, coronary artery disease; PRS, polygenic risk score.

Supplemental Figure 4. Forest plot for cardiovascular disease according to genetic risk for type 2 diabetes and lifestyle behavior.

Generic risk for type 2 diabetes	Lifestyle subgroups	No. of event/Total No.	Absolute risk (%)	Adjusted HR (95% CI)	*P* value	*P* for interaction
						0.239
Low genetic risk	Favorable	1830/38009	4.82	Ref		
	Intermediate	1386/21769	6.37	1.34 (1.25-1.43)	<0.001	
	Unfavorable	636/6805	9.35	2.05 (1.87-2.24)	<0.001	
Intermediate genetic risk	Favorable	5625/109760	5.13	1.08 (1.02-1.14)	0.005	
	Intermediate	4564/66812	6.83	1.46 (1.39-1.54)	<0.001	
	Unfavorable	2178/23110	9.42	2.08 (1.96-2.22)	<0.001	
High genetic risk	Favorable	1856/33562	5.53	1.17 (1.09-1.25)	<0.001	
	Intermediate	1578/21512	7.34	1.59 (1.48-1.70)	<0.001	
	Unfavorable	775/8069	9.61	2.14 (1.97-2.33)	<0.001	
Very high genetic risk	Favorable	103/1660	6.21	1.34 (1.09-1.63)	0.004	
	Intermediate	91/1183	7.69	1.72 (1.39-2.13)	<0.001	
	Unfavorable	54/466	11.59	2.69 (2.05-3.52)	<0.001	

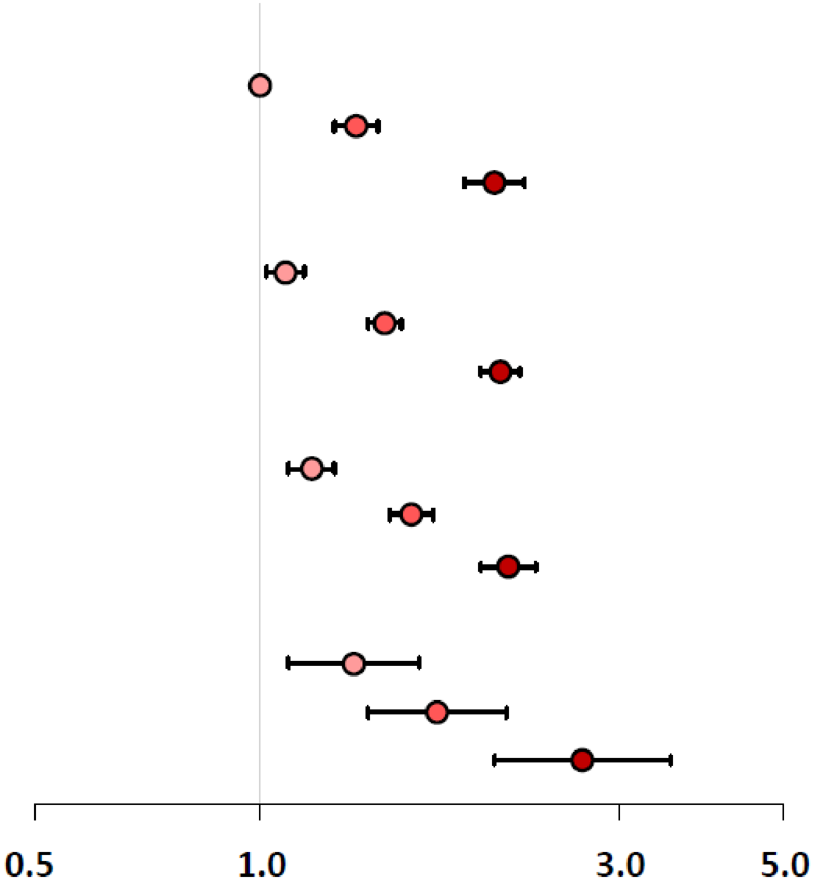

Cox regression model were adjusted for age, sex, genotyping array, first ten principal components of ancestry.

P for interaction is for testing the interaction between four genetic risk groups and three lifestyle categories.

Abbreviations: HR, hazard ratio; CI, confidence interval.

Supplemental Figure 5. Forest plot for cardiovascular disease of age interaction according to genetic risk for type 2 diabetes and lifestyle behavior using reference group as unfavorable lifestyle.

Age group	T2D PRS subgroup	Lifestyle subgroup	No. of Event/ Total No.	Absolute risk (%)	Adjusted HR (95% CI)	*P* value	*P* for interaction
							<0.001
40 to 50 years	High genetic risk	Unfavorable	130/2373	5.48	Ref		
	(Top 20 percentile)	Intermediate	211/6250	3.38	0.63 (0.51-0.79)	<0.001	
		Favorable	205/9623	2.13	0.40 (0.32-0.50)	<0.001	
	Low genetic risk	Unfavorable	384/8185	4.69	Ref		
	(Bottom 80 percentile)	Intermediate	672/23782	2.83	0.61 (0.54-0.70)	<0.001	
		Favorable	736/40383	1.82	0.41 (0.36-0.46)	<0.001	
51 to 69 years	High genetic risk	Unfavorable	699/6162	11.34	Ref		
	(Top 20 percentile)	Intermediate	1458/16445	8.87	0.75 (0.69-0.83)	<0.001	
		Favorable	1754/25599	6.85	0.58 (0.53-0.63)	<0.001	
	Low genetic risk	Unfavorable	2430/21730	11.18	Ref		
	(Bottom 80 percentile)	Intermediate	5278/64799	8.15	0.70 (0.67-0.74)	<0.001	
		Favorable	6719/107386	6.26	0.53 (0.50-0.55)	<0.001	

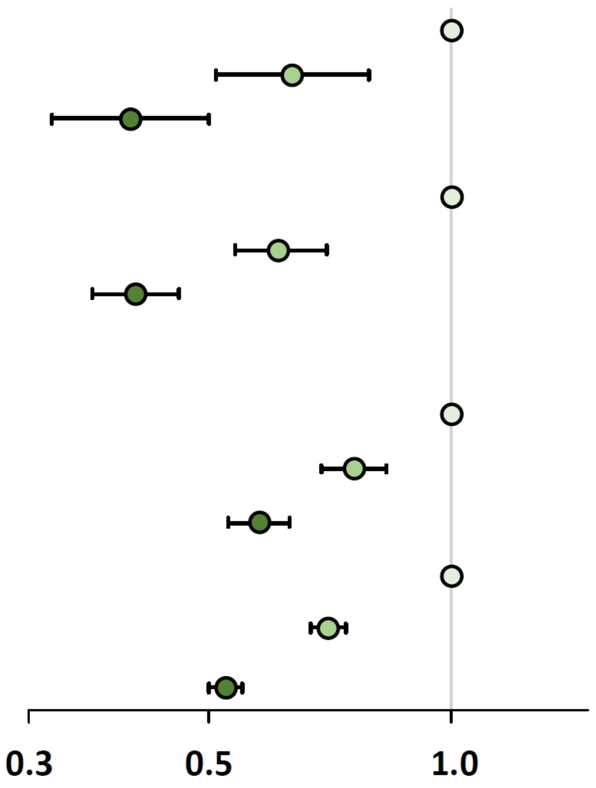

Cox regression model were adjusted for age, sex, genotyping array, first ten principal components of ancestry.

P for interaction is for testing the interaction between each genetic risk by lifestyle category and age subgroups.

Abbreviations: HR, hazard ratio; CI, confidence interval.

Supplemental Figure 6. Forest plot for cardiovascular disease of age interaction according to genetic risk for type 2 diabetes and metabolic health status using reference group as metabolically unhealthy subgroup.

Age group	T2D PRS subgroup	MetS subgroup	No. of Event/ Total No.	Absolute risk (%)	Adjusted HR (95% CI)	*P* value	*P* for interaction
							0.003
40 to 50 years	High genetic risk	3 components	213/4193	5.08	Ref		
	(Top 20 percentile)	2 components	128/3992	3.21	0.63 (0.51-0.79)	<0.001	
		0 or 1 component	158/8236	1.92	0.40 (0.32-0.50)	<0.001	
	Low genetic risk	3 components	605/13239	4.57	Ref		
	(Bottom 80 percentile)	2 components	447/15057	2.97	0.61 (0.54-0.70)	<0.001	
		0 or 1 component	629/36597	1.72	0.41 (0.36-0.46)	<0.001	
51 to 69 years	High genetic risk	3 components	1602/15104	10.61	Ref		
	(Top 20 percentile)	2 components	1040/12387	8.40	0.75 (0.69-0.83)	<0.001	
		0 or 1 component	985/16060	6.13	0.58 (0.53-0.63)	<0.001	
	Low genetic risk	3 components	5253/10.32	10.32	Ref		
	(Bottom 80 percentile)	2 components	3858/49878	7.74	0.70 (0.67-0.74)	<0.001	
		0 or 1 component	4189/74452	5.63	0.53 (0.50-0.55)	<0.001	

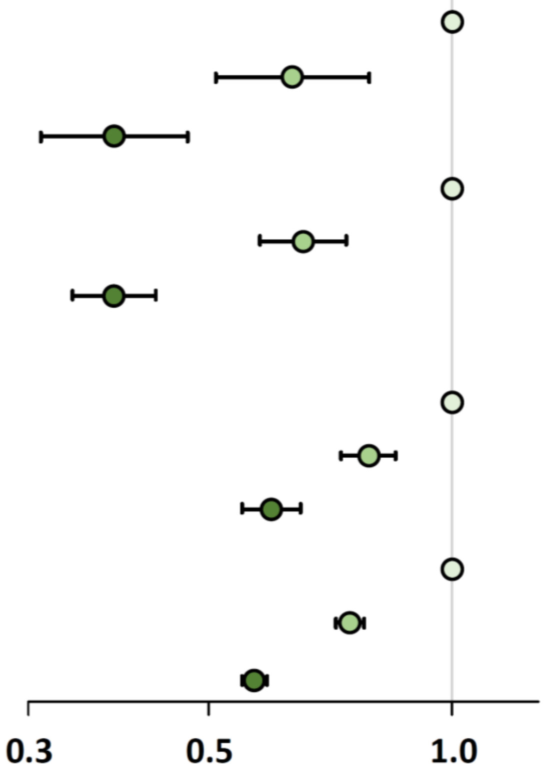

Cox regression model were adjusted for age, sex, genotyping array, first ten principal components of ancestry.

P for interaction is for testing the interaction between genetic risk by metabolic health category and age subgroups.

Abbreviations: T2D, type 2 diabetes; PRS, polygenic risk score; MetS, metabolic syndrome; HR, hazard ratio; CI, confidence interval.
